# Under the skin: *Ixodes* ticks in the subcutaneous tissue of red foxes (*Vulpes vulpes*) from Germany

**DOI:** 10.1186/s13071-020-04061-x

**Published:** 2020-04-21

**Authors:** Maja Haut, Nina Król, Anna Obiegala, Johannes Seeger, Martin Pfeffer

**Affiliations:** 1 Institute of Animal Hygiene and Veterinary Public Health, Faculty of Veterinary Medicine, University of Leipzig, An den Tierkliniken 1, 04103 Leipzig, Germany; 2 Institute of Anatomy, Histology and Embryology, Faculty of Veterinary Medicine, University of Leipzig, An den Tierkliniken 43, 04103 Leipzig, Germany

**Keywords:** Ectoparasites, Subcutaneous, *Ixodes* spp., Tick, Red fox, Germany, Europe

## Abstract

**Background:**

*Ixodes* spp. are vectors of zoonotic pathogens. All three active life stages (larvae, nymphs, adults) need to feed on a host in order to develop. Usually ticks parasitize attached to the external surface of their hosts’ skin. Interestingly, in some cases ticks can also be found in the subcutaneous tissue in a variety of hosts, such as red foxes (*Vulpes vulpes*), raccoon dogs (*Nyctereutes procyonoides*) and dogs.

**Methods:**

The visceral side of 126 red fox-furs from Germany was examined visually searching for ticks. The localization of ticks was recorded and assigned to ten specific body parts. Morphological identification of ticks was performed according to standardized taxonomic protocols. Ticks which could not be further identified were examined genetically *via* conventional PCR targeting the *16S* rRNA and *cox*1 gene. Hematoxylin and eosin (H&E) staining was used for histopathological examination.

**Results:**

In 111 out of 126 (88.1%) examined coats, at least one tick was found in the subcutaneous tissue. A total of 1203 ticks were removed from the subcutaneous tissue. Well-preserved ticks could be identified based on morphological criteria, but most ticks were in a progressed state of decomposition. Here, morphological species identification was not successful. Also, PCR methods did not lead to a successful species identification. The following species and development stages were found by morphological identification: *Ixodes ricinus* (female, *n* = 289; male, *n* = 8; nymph, *n* = 1), *I. hexagonus* (female, *n* = 2), *I. canisuga* (female, *n* = 1). Male *I. ricinus* were found individually or copulating in pairs with females. Subcutaneous ticks were localized at three predominant affected body parts: ears, axillar and inguinal region. Histological examination of subcutaneous ticks revealed a granulomatous panniculitis.

**Conclusions:**

To the authors’ knowledge, this is the first finding of highly prevalent subcutaneous ticks in red foxes from Germany. Subcutaneous location of ticks seems to be very common in red foxes and the rule rather than the exception. Deep embedment of longirostra and long feeding times of females seem to put the subcutaneous location in favor. Most foxes were infested in the inguinal area, where the skin is thin and less hairy.
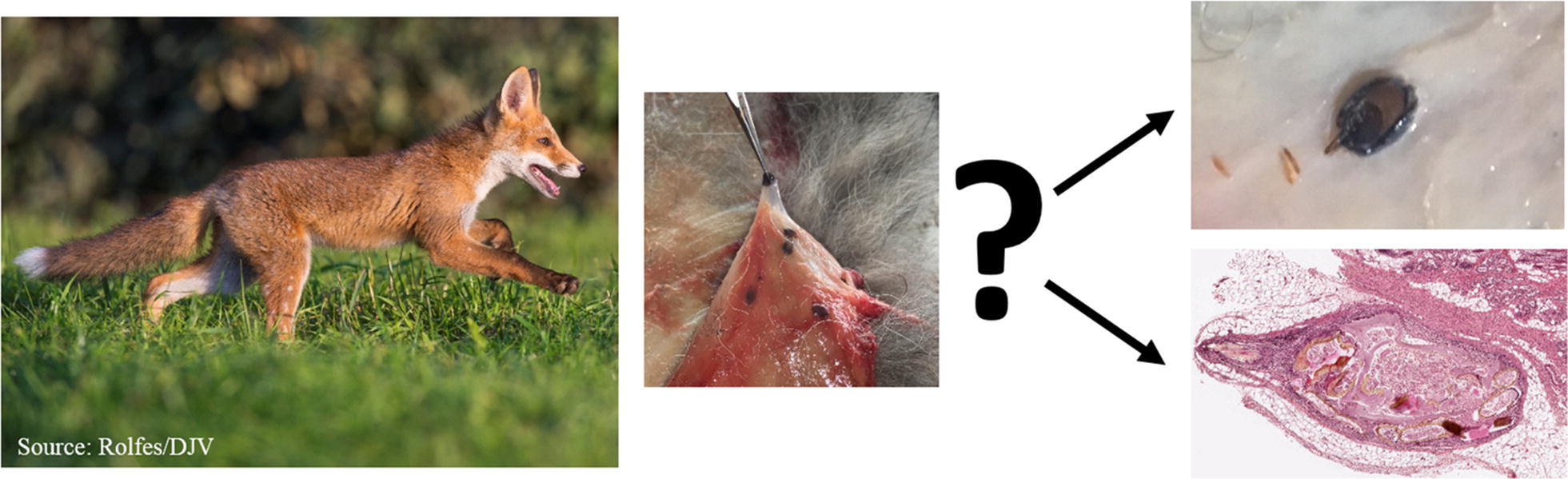

## Background

Ticks belonging to the genera *Ixodes* and *Dermacentor* (Family: Ixodidae) are the most prevalent in Europe [1]. All active tick life stages (six-legged larva, eight-legged nymph and females) require blood meals from hosts for molting (immatures) or for oviposition (females) with the exception of males which feed facultatively and are localized on hosts mainly for mating purposes [1, 2].

In Germany the species *Ixodes ricinus*, *I. hexagonus*, *I. canisuga* and *I. kaiseri* have been frequently collected from foxes [3–8]. Additionally, *Dermacentor reticulatus* and *Haemaphysalis concinna* were occasionally found attached to red foxes’ skin [7, 9]. Ticks are usually strongly attached to the external surface of their hosts to remain in place for a blood meal. Thereby tick’ mouthparts penetrate different skin layers, depending on their length. Ticks with long mouthparts (longirostra e.g. *Amblyomma*, *Hyalomma* and *Ixodes*) embedded into deep layers of the dermis while ticks with short mouthparts (brevirostra e.g. *Dermacentor*, *Haemaphysalis* and *Rhipicephalus*) barely penetrate the epidermis [10, 11]. *Ixodes ricinus* larvae are attached for 2–4 days, nymphs for 4–6 days and females for 6–10 days [12].

Interestingly, in some cases ticks can also be found in the subcutaneous tissue in a variety of hosts including red foxes. The presence of subcutaneous ticks such as *I. ricinus*, *I. hexagonus*, *I. crenulatus* and *D. reticulatus* was noted in red foxes in several case studies from the UK, Poland, Austria, Romania, Slovakia and Czech Republic [13–20]. In the USA, the native tick species *Amblyomma americanum* was likewise discovered in the subcutaneous tissue of red foxes [21].

Besides red foxes, single case reports of subcutaneous ticks were previously recorded in raccoon dogs (*Nyctereutes procyonoides*) from Poland [22], in a dog from Sweden [23] and in a child from South Korea [24].

Hitherto, small sample sizes were reported in most cases also rather as occasional findings [13–17, 21]. Thus far, no published data exist about subcutaneous ticks in red foxes or other animals from Germany. This is why this study is aiming to evaluate the frequency of subcutaneous ticks in red foxes from Germany using a large number of samples. The localization of subcutaneous ticks at different body regions of the red fox has not been analyzed yet; therefore, we documented the localization of each tick in order to determine the abundance of these ticks in the subcutaneous tissue. Precise species identification of subcutaneous ticks based on morphological criteria is possible only to a limited extent due to the advanced degradation process in subcutaneous tissue, as seen in previous studies [18]. Hence, this study aims to use molecular biology techniques to identify subcutaneous ticks to species level.

## Methods

### Animal collection

Between November 2018 and February 2019 local hunters shot red foxes and other wild game in course of traditional hunting. In cooperation with a scrape station (Fellwechsel Berlin, Germany), local hunters from all over Germany deposited fox carcasses in plastic bags labelled with the date and exact geographical location in deep-freezers. Only adult foxes with good nutritional status were selected by hunters. Fox cadavers were stored at − 80 °C until flaying. For enabling the histological studies one fox was skinned directly after hunting.

### Tick collection and identification

**Fig. 1:**
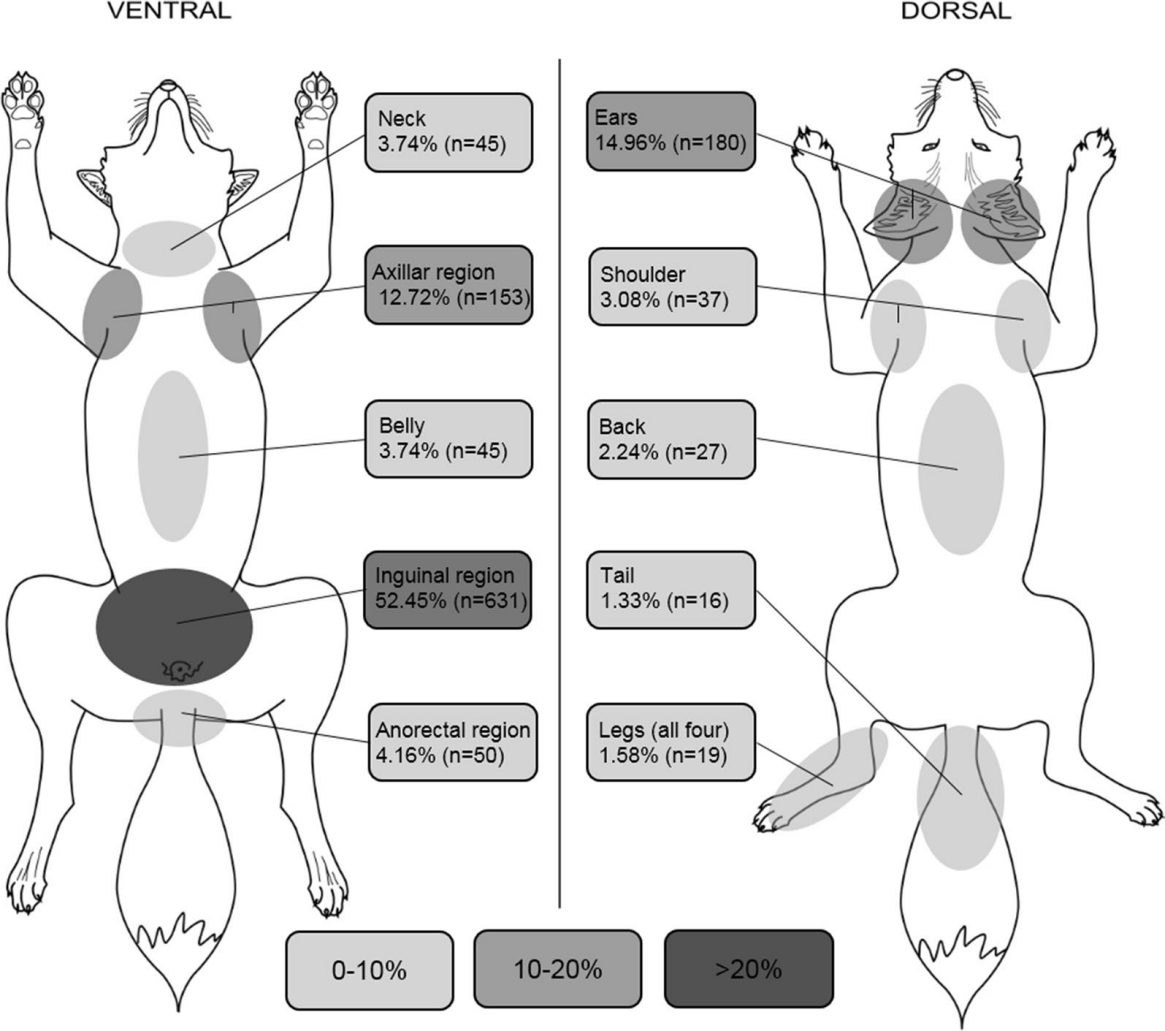
Density of ticks expressed as percentage of total number of ticks depending on body region (*n* = 111 red foxes). A darker grey in the figure indicates a higher density of ticks

**Fig. 2:**
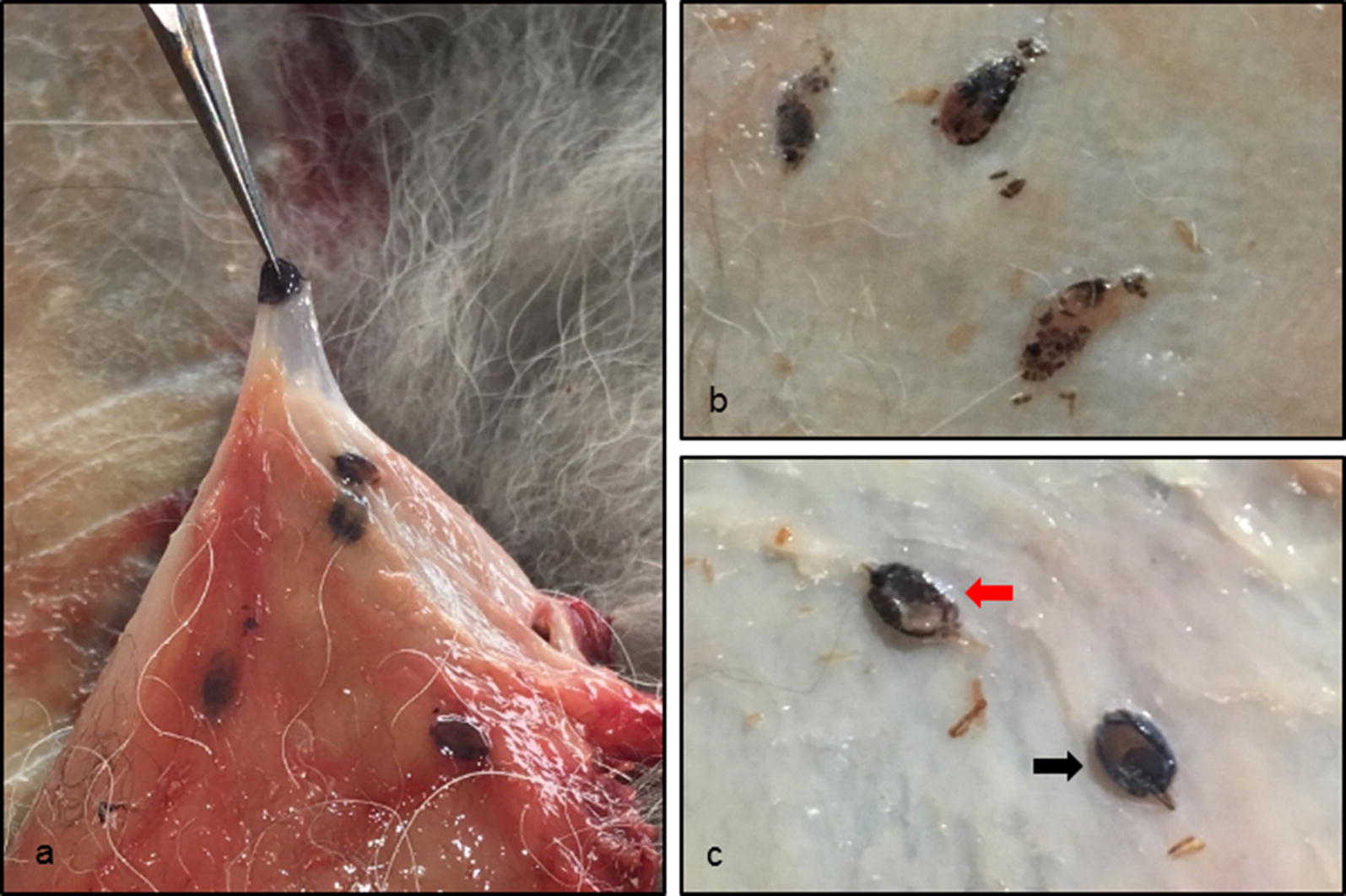
Macroscopic view on ticks with subcutaneous localization. **a** One tick lifted up with a fine-tipped forceps and other ticks in the surrounding tissue. **b** Ticks in advanced stages of decomposition. **c** Well-preserved ticks in dorsal (black arrow) and ventral positon (red arrow)

The visceral side of each fur was examined visually for the presence of subcutaneous ticks. The localization of ticks was recorded on a pre-drawn schematic picture and assigned to ten specific body parts (ears, neck, axillar region, shoulder, back, belly, inguinal region, anorectal region, legs and tail) in order to create a body map of tick density (Fig. 1), as recommended by Lydecker et al. [25]. Afterwards, the ticks were individually dissected from the subcutaneous tissue with fine-tipped forceps and a scalpel and stored at − 20 °C (Fig. 2). Well-preserved ticks could be identified based on morphological criteria according to standardized taxonomic protocols [26] under a binocular at 50× magnification (Motic SMZ-171, Motic Deutschland GmbH, Wetzlar, Germany).

### DNA extraction and PCR methods

Ticks which could not be morphologically identified were further processed by conventional PCR to identify them to species level. For the DNA extraction, 1 g of steel beads (sized 2.8 mm) and 500 µl of PBS were added to each tick. The samples were then homogenized at 5000× *rpm* for 2 × 15 s with 15 s break intervals in a Precellys®24 tissue homogenizer (Bertin Technologies, Montigny Le Bretonneux, France).

The genomic DNA was extracted using the QIAamp DNA Mini Kit (Qiagen, Hilden, Germany) following the manufacturer’s instructions. PCR amplifications of partially cytochrome *c* oxidase subunit 1 (*cox*1) gene (710 bp) and of the *16S* ribosomal ribonucleic acid (rRNA) gene (455 bp) were performed using previously published PCR protocols [8, 27]. PCR products were visualized by gel electrophoresis on 2% agarose gels stained with HDGreen Plus DNA Stain (Intas Science Imaging Instruments GmbH, Göttingen, Germany).

### Histological examination of subcutaneous tissue

Subcutaneously located ticks were removed with the skin and the surrounding tissue from the complete fur and immediately fixed in 10% buffered formalin, dehydrated in ascending ethanol series (20–100%) and embedded in paraffin blocks. Seven-micron thick sections were cut with a rotary microtome (Leica RM 2155, Leica Microsystems Nussloch GmbH, Nussloch, Germany), placed on glass slides and stained with hematoxylin and eosin (H&E). Microscopy and pictures were performed using a Zeiss Axioplan 2 imaging (Carl Zeiss Microscopy, Jena, Germany) equipped with an objective 25×/0.8 Plan-Neofluar Oil and a color camera ProgRes C14 (Jenoptik, Jena, Germany). Whole section overviews were obtained by tile scans with a 25×/0.8 Plan-Neofluar Oil objective using a tile scan routine under Openlab 5.5 (Improvision, Coventry, UK). During histological examination it was noticed that the inflammatory response was related to the stage of degradation of the tick. Therefore, the ticks were divided into 3 categories: (i) well-preserved ticks with correct position of hypostome and the appendages and intact exoskeleton and body; (ii) deformed ticks with intact exoskeleton; and (iii) ticks with broken, fully deformed exoskeleton.

### Statistical analysis

Confidence intervals (95% CI) for prevalence rates of subcutaneous ticks and their life stage were determined by the Clopper & Pearson method with GraphPad Software (GraphPad Software Inc., San Diego, CA, USA). The Mann-Whitney U-test with a significance level of *α* = 0.05 was used to compare the different body regions. The independence of compared small sample sizes (*n* < 30) was tested with Fisher’s exact test. Statistical analyses were performed using IBM SPSS Statistics for Windows, Version 25.0 (IBM Corp, Armonk, NY, USA).

## Results

### Animal collection

For the present study 126 red foxes (75 males and 51 females), were collected and examined from central [federal states of Brandenburg (*n* = 1); Hessen (*n* = 2); North Rhine-Westphalia (*n* = 56): Saxony (*n* = 3); and Thuringia (*n* = 5)] and northern Germany [federal states of Lower Saxony (*n* = 45); and Schleswig-Holstein (*n* = 14)].

### Tick collection

**Fig. 3:**
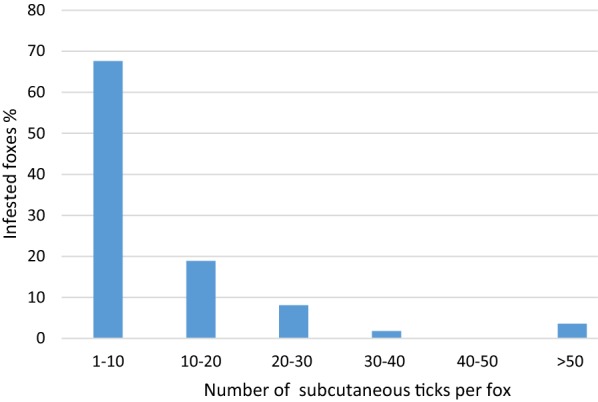
The distribution of tick burden diverged between 1 to 79, a total of 1203 ticks could be removed. Most of the foxes were infested with only a small number of ticks

In 111 out of 126 (88.1%, 95% CI: 81.13–93.18%) examined coats, at least one tick was found in the subcutaneous tissue. The frequency of the occurrence of subcutaneous ticks between male and female foxes did not differ significantly (*P* < 0.590). The tick burden per fox varied from 1 to 79, with a mean of 10.8 (SD = 14.02). Most of the foxes (69.0%) were infested with a maximum of 10 ticks (Fig. 3). A total of 1203 ticks were removed from the subcutaneous tissue. Morphological features of the Ixodidae, like the presence of a dorsal scutum or mouthparts anteriorly attached and visible dorsally, were present in all ticks found. Most ticks (*n* = 902; 75%) were in a progressed state of decomposition and the developmental stage as well as the species level could not be determined. However, specific criteria were met to assign the ticks to the family Ixodidae. In these cases, species identification using molecular biological methods was not successful. Well-preserved ticks (*n* = 301; 25.0%) could be identified to the species level based on morphological criteria. The following species and developmental stages were found (Table 1): *Ixodes ricinus* (female, male, nymph); *I. hexagonus* (female); *I. canisuga* (female). The most prevalent life stage were females (97.0%; *n* = 292/301, 95% CI: 94.40–98.62%) followed by males (2.7%; *n* = 8/301, 95% CI: 1.15–5.17%) and nymphs (0.3%; *n* = 1/301, 95% CI: 0.01–1.84%). Females were identified most frequently as *I. ricinus* (99.0%; *n* = 289/292, 95% CI: 97.03–99.79%). All male ticks belonged to *I. ricinus* and were mainly found as mating pairs with females (*n* = 6/8), but also individually (*n* = 2/8).

**Table 1:** Tick species and life stages of well-preserved ticks found in the subcutaneous tissue

Tick species	No. of ticks (%)	No. of males	No. of females	No. of nymphs
*I. ricinus*	298 (99.0)	8	289	1
*I. hexagonus*	2 (0.7)		2	
*I. canisuga*	1 (0.3)		1	
Total (%)	301 (100)	8 (2.7)	292 (97.0)	1 (0.3)

### Subcutaneous localization of ticks

Subcutaneous ticks were localized at all 10 defined body parts. Significantly, most ticks were found in the inguinal area compared to all other body regions (*U*_(2268)_ = 262431.00, *Z* = − 26.629, *P* < 0.001). Overall, 52.5% (*n* = 631/1203) of all collected ticks were found in this region, distributed among 91 out of 111 infested foxes. Compared to all other regions most foxes were infested with subcutaneous ticks in the inguinal area (*P* < 0.0059 to *P* < 0.0001; Table 2). The second and third most common body region were the areas around the ears and the axilla. In total, 80.2% (*n* = 964) of all collected ticks were observed in these three body regions (Fig. 1). Further regions showed a significantly lower burden of subcutaneous ticks (*U*_(5292)_ = 2072521.50, *Z* = − 30.909, *P* < 0.001). The difference in the distribution of subcutaneous ticks between female and male foxes was not statistically significant for the most frequently infested body parts (inguinal region: *U*_(126)_ = 1711.00, *Z* = − 1.015, *P* = 0.310; axillar region: *U*_(126)_ = 1869.00, *Z* = − 0.274, *P* = 0.784; ears *U*_(126)_ = 1743.50, *Z* = − 0.889, *P* = 0.374).

**Table 2:** In the majority of fox furs subcutaneous ticks were found in more than one body region. But significantly most foxes were infested in the inguinal region in a direct paired comparison with all other body regions

Body part	No. of infested foxes (%)
Ears	69/126 (54.8)**
Neck	28/126 (22.2)***
Axillar region	35/126 (27.8)***
Shoulder	20/126 (15.9)***
Back	13/126 (10.3)***
Belly	21/126 (16.7)***
Inguinal region	91/126 (72.2)
Anorectal region	18/126 (14.3)***
Legs	13/126 (10.3)***
Tail	6/126 (4.8)***

*P ≤ 0.05, **P ≤ 0.01, ***P ≤ 0.001

### Histological studies of subcutaneous tissue

**Fig. 4:**
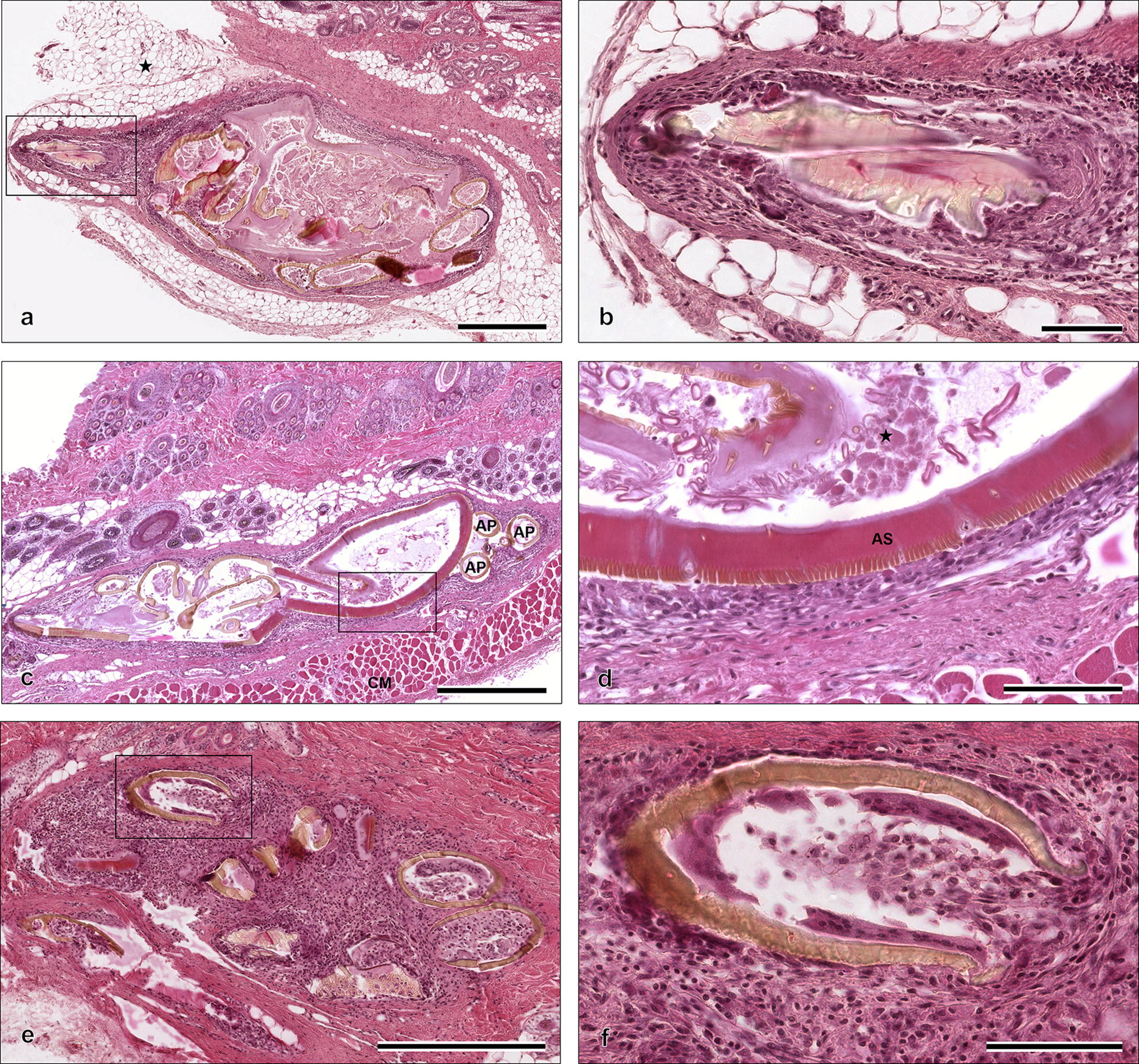
Histological examination of ticks located in the subcutaneous tissue of a red fox from Germany. **a** Well-preserved tick of category 1 in the subcutaneous fat tissue (marked by asterisk) (H&E). **b** The hypostome of the tick from **a** at a higher magnification. The hypostome is surrounded by a mixture of inflammatory cells and a fibrous capsule (H&E). **c** A deformed tick of category 2 in the subcutaneous tissue above the cutaneous muscle (*panniculus carnosus*) (CM). Three appendages (AP) are visible, but due to high deformation of the tick not assignable to legs or palpi (H&E). **d** Magnification of alloscutum (AS), the inner part of the tick and the surrounding inflammation of the tick from **c**. The inner part of the tick contains of cell debris (marked by asterisk) without any cellular structure (H&E). **e** The broken parts of exoskeleton of category 3 tick (H&E). **f** Infiltration of broken exoskeleton with inflammatory cells consisting of histiocytes, eosinophils and multinuclear giant cells (H&E). *Scale-bars*: **a**, **c**, **e**, 500 µm; **b**, **d**, **f**, 100 µm

In total, 23 subcutaneous ticks collected from one fox were histologically examined (Fig. 4). Only one tick was assigned to category 1, 11 belonged to category 2, and 11 to category 3. Ticks of category 1 were surrounded by a moderate granulomatous inflammation of subcutaneous fat tissue (panniculitis) consisting of histiocytes, macrophages, multinuclear giant cells and eosinophils. Neutrophils were only occasionally seen. A mild dermal inflammatory response was present with mild exocytosis of inflammatory cells in the epidermis. Histiocytes, macrophages and eosinophils were the most common cells observed. Additionally, moderate epidermal inter- and intracellular edema and moderate lamellar orthokeratotic hyperkeratosis were found. Intradermal and intracorneal pustules filled mainly with neutrophils and rarely with macrophages were present.

A high degree of deformation of ticks was visible in most of the histological sections. The presence of ticks of category 2 resulted in a mild granulomatous panniculitis composed of histiocytes, macrophages, multinuclear giant cells, eosinophils and sparse neutrophils.

In the inner part of the ticks of category 1 and 2 no soft tissue of the arthropod could be identified, and no cellular structures were recognizable. The broken exoskeleton of ticks belonging to category 3 was filled with a mixed inflammatory cell infiltrate consisting of histiocytes, macrophages and multinuclear giant cells. These ticks were surrounded by a severe granulomatous panniculitis without inflammatory changes of dermis and epidermis.

Histological examination of ticks with surrounding tissue and skin confirmed the subcutaneous localization. Increasing eosinophilic granulomatous inflammation from category 1 to 3 were observed. During histopathological examination no blood degradation products were observed.

## Discussion

Foxes are important hosts for a variety of endo- and ectoparasites and thereby reservoirs for animal pathogens and zoonoses [28]. The observed prevalence of subcutaneous ticks (88.1%) in the present study is higher than in Poland (38%) [20] and Czech Republic/Romania (15.4%) [18]. Moreover, the prevalence of subcutaneous ticks is higher in comparison with external tick prevalence in red foxes from Italy (7.4%) [29] and Romania (53.9%) [30], but coincides to studies from Spain (51.1–84.6%) [31–33] and Germany (76.5–82.6%) [7, 34]. Additionally, the infestation intensity of subcutaneous ticks (mean = 10.8) in the present study is similar to external ticks from another study examining 1268 foxes in Germany (mean = 11.7) [34]. In that study, 50.9% of foxes were infested with a maximum of 5 ticks on their external surface, while in the present study 43.2% of the foxes were infested with a maximum of 5 ticks. However, in some cases both subcutaneous and external ticks were collected numerously from a few foxes (3.6–5.7%) with more than 50 ticks per fox [7].

In Europe, several ticks of the genera *Ixodes*, *Dermacentor*, *Haemaphysalis*, *Hyalomma* and *Rhipicephalus* are known to parasitize red foxes [7, 8, 29, 31, 32]. In Germany, *I. ricinus*, *I. hexagonus*, *I. canisuga* and *I. kaiseri* were commonly collected from foxes, while *D. reticulatus* and *H. concinna* were occasionally found [7, 9]. Accordingly, we found three of the previously mentioned longirostral species (*I. ricinus*, *I. hexagonus* and *I. canisuga*) in the subcutaneous tissue. Likewise, previously published data from USA and Czech Republic/Romania show regional differences of tick species in the subcutaneous tissue depending on the prevailing tick fauna [18, 20, 21].

Longirostra (e.g. *Amblyomma*, *Hyalomma* and *Ixodes*) embed more deeply to the hosts skin in comparison to brevirostra (e.g. *Dermacentor*, *Haemaphysalis* and *Rhipicephalus*) which attach more superficially [10, 11]. In the current study, exclusively *Ixodes* ticks were collected from the subcutaneous tissue of the foxes. These findings are in line with published records of subcutaneous ticks, where *Ixodes* ticks dominated over *Dermacentor* spp. which were found only occasionally [18, 20]. The deep embedment (as in longirostra) was previously discussed to be favorable for the subcutaneous location of ticks [18]. Our findings are in line with this assumption as longirostra were significantly more often found subcutaneously in comparison to brevirostra.

The feeding period of ixodid ticks depends on the tick’s developmental stage and varies between several days (immatures) up to two weeks (females) [12]. D’Amico et al. [18] highlighted that a long feeding duration is putting ticks in favor of being found in the subcutaneous tissue. In agreement with this, significantly more females were found subcutaneously (97.0%; *P* < 0.0001) when compared to males and nymphs in the current study. Male *I. ricinus* are feeding facultatively, taking small blood meals and remain on the host searching for females to mate [1]. Facultative feeding may explain why only two males were found individually in subcutaneous location. It cannot be ruled out that copulating males are separated from females during degradation process or preparation of ticks. Male *Ixodes* can remain in copulation for 6–11 days throughout the feeding periods of female *Ixodes* [35], which may also explain why *I. ricinus* mating pairs are found in the subcutaneous tissue. Rare findings of nymphs, and none of larvae, may be related to their shorter hypostome. Additionally, a shorter feeding duration of immature ticks may explain their absence. So far, the duration and mechanism causing the subcutaneous localization of ticks are unknown, but long feeding times of females seems to be crucial for penetration.

In this study, subcutaneous ticks were usually located at three predominant body parts: ears, axillar and inguinal area, which is in line with previous studies. Smith et al. [21] reported subcutaneous ticks in the inguinal, axillar and perianal region, while Drozdz [14] found ticks in subcutaneous location in the area of ears, shoulder and belly. Neither of these reports provide information about the frequency of occurrence. The present study identified the most significant infested body parts of red foxes. Strikingly, these body parts are less hairy, folded and the skin is thin. This is in concordance with ESCCAP Guidelines Control of Ectoparasites in Dogs and Cats, which generally found the same preferences for tick attachment in dogs and cats [36].

So far, the subcutaneous presence of ticks was mostly reported from red foxes [13–21] Only single case reports of subcutaneous ticks in raccoon dogs and from one single domestic dog exists at the moment [22, 23] In a study inspecting 134 roe deer furs from inside and outside, only external ticks were found and none in the subcutaneous tissue [37]. Thus far, little is currently known about this phenomenon, leaving questions concerning the mechanisms as to how ticks become subcutaneous and which animals can be affected.

Histological examination revealed that the quality of the inflammatory response is related to the stage of degradation of the tick. All subcutaneous ticks were surrounded by granulomatous panniculitis, these findings agree with D’Amico et al. [18]. When the exoskeleton was intact, the inflammation was mild-moderate. But in case of broken exoskeletons a severe inflammatory response was present. The one well-preserved tick showed an acute and local mechanical irritation of dermis and epidermis. This could be a sign that this tick has recently penetrated the skin. The inner part of the ticks’ intact exoskeleton contained a mixture of cell debris and no cellular structure was recognizable, which may explain why DNA isolation was not successful.

## Conclusions

Subcutaneous location of ticks seems to be very common in red foxes and is rather the rule than the exception. They were predominantly located at less hairy, thin and folded skin body parts of the fox. The unusual tick location is not linked to one tick species or development stage, however, mainly female longirostra were found. Decisive factors for the subcutaneous location of ticks appear to be the deep embedment of longirostra and long duration time of attachment of females. Further examinations are needed to evaluate the mechanism leading to the subcutaneous localization of ticks. Nevertheless, this phenomenon is not an evolutionary advantage for ticks, as all collected ticks were dead and at various stages of decomposition.

## Data Availability

The data supporting the conclusions of this article are included within the article. The raw data used and/or analyzed during the present study are available from the corresponding author upon reasonable request.

## Abbreviations

CI: Confidence interval
*cox*1: Cytochrome *c* oxidase subunit 1
DNA: Deoxyribonucleic acid
H&E: Hematoxylin and eosin
PBS: Phosphate-buffered saline
PCR: Polymerase chain reaction
Rpm: Revolutions per minute
rRNA: Ribosomal ribonucleic acid
SD: Standard deviation
